# Spatial Clustering of County-Level COVID-19 Rates in the U.S.

**DOI:** 10.3390/ijerph182212170

**Published:** 2021-11-19

**Authors:** Marcus R. Andrews, Kosuke Tamura, Janae N. Best, Joniqua N. Ceasar, Kaylin G. Batey, Troy A. Kearse, Lavell V. Allen, Yvonne Baumer, Billy S. Collins, Valerie M. Mitchell, Tiffany M. Powell-Wiley

**Affiliations:** 1 Department of Health Behavior and Health Education, School of Public Health, University of Michigan, 1450 Washington Heights, Ann Arbor, MI 48109, USA; marcusan@umich.edu (M.R.A.); jnbest@umich.edu (J.N.B.); 2 Neighborhood Social and Geospatial Determinants of Health Disparities Laboratory, Population and Community Health Sciences Branch, Intramural Research Program, National Institute on Minority Health and Health Disparities, National Institutes of Health, Bethesda, MD 20892, USA; kosuke.tamura@nih.gov; 3 Department of Medicine, Internal Medicine-Pediatrics Residency, Johns Hopkins University, 251 Bayview Boulevard, Baltimore, MD 21224, USA; joniqua@jhmi.edu; 4 College of Medicine, University of Kentucky, 800 Rose Street MN 150, Lexington, KY 40506, USA; kaylind.batey@yahoo.com; 5 Department of Psychology, Howard University, 525 Bryant Street, NW, Washington, DC 20059, USA; troy.kearse@bison.howard.edu; 6 Department of Public Health, University of New England, 11 Hills Beach Road, Biddeford, ME 04005, USA; lavell.allen27@gmail.com; 7 Social Determinants of Obesity and Cardiovascular Risk Laboratory, Cardiovascular Branch, Division of Intramural Research, National Heart, Lung, and Blood Institute, National Institutes of Health, Bethesda, MD 20892, USA; yvonne.baumer@nih.gov (Y.B.); billy.collins@nih.gov (B.S.C.); valerie.mitchell@nih.gov (V.M.M.); 8 Adjunct Investigator, Intramural Research Program, National Institute on Minority Health and Health Disparities, National Institutes of Health, Bethesda, MD 20892, USA

**Keywords:** COVID-19, geographic information systems, applied spatial statistics

## Abstract

Despite the widespread prevalence of cases associated with the coronavirus disease 2019 (COVID-19) pandemic, little is known about the spatial clustering of COVID-19 in the United States. Data on COVID-19 cases were used to identify U.S. counties that have both high and low COVID-19 incident proportions and clusters. Our results suggest that there are a variety of sociodemographic variables that are associated with the severity of COVID-19 county-level incident proportions. As the pandemic evolved, communities of color were disproportionately impacted. Subsequently, it shifted from communities of color and metropolitan areas to rural areas in the U.S. Our final period showed limited differences in county characteristics, suggesting that COVID-19 infections were more widespread. The findings might address the systemic barriers and health disparities that may result in high incident proportions of COVID-19 clusters.

## 1. Introduction

Since late 2019, coronavirus disease 2019 (COVID-19), caused by the novel severe acute respiratory syndrome coronavirus-2 (SARS-CoV-2), has rapidly spread around the globe [1,2,3]. Early studies suggested that SARS-CoV-2 originated in a local market in China, where it was transmitted from animals to humans [4]. Initial COVID-19 cases were reported in November 2019, with the World Health Organization (WHO) declaring it a pandemic on 11 March 2020 [5]. Emerging research suggests human-to-human transmission of COVID-19 through respiratory droplets or direct contact with an infected person [1,3,4,6,7]. Based on data from August of 2021, there were over 202 million global COVID-19 cases, with over four million deaths globally [8]. The highest percentage of cumulative cases associated with the COVID-19 burden was concentrated in the Americas, Europe, and Asia [8].

The spread of COVID-19 within the United States (U.S.) may be influenced by sociodemographic conditions that vary geographically [9,10,11,12,13]. For example, initial U.S. reports identified geographic disparities in the availability of personal protective equipment, ventilators, intensive care unit beds, hospital beds, and other vital medical resources necessary to treat COVID-19 [9,14,15]. As more data have become publicly available [16,17], racial/ethnic disparities in COVID-19 incident proportions and associated fatalities within the U.S. are likely attributable to the intersection of long-standing social injustices and structural discrimination with socioeconomic status (S.E.S.) and built (or physical) environmental factors. Many of these factors unequally distribute the risk for COVID-19 across the U.S. [18,19]. Furthermore, urban areas may be important to study with regard to the COVID-19 pandemic. For example, those living in urban areas are more likely to be subjected to racial and socioeconomic residential segregation, which can inequitably expose residents to a cadre of factors that would increase the overall transmission of the virus, as well as the overall severity of the disease [20].

Some county-level characteristics may be correlated with COVID-19. Recent studies on COVID-19 focusing on neighborhood social contexts demonstrated that poverty, comorbidities, and race/ethnicity are some of the important correlates to COVID-19 outcomes [7]. Other studies showed that living in poverty may increase the risk for contracting COVID-19 through impaired access to healthcare [21,22], higher risk of comorbidities [23], and an impaired ability to practice physical or social distancing [24]. Specifically, using cross-sectional data within the United States, researchers have examined the relationship between county-level sociodemographic risk factors on COVID-19 incidence and mortality. Their analyses suggest that increases in county level social vulnerability indices, especially county-level minority proportions and English language proficiency, were related to increases in both incidence and mortality rates [25]. These findings have been supported by other articles using preliminary data from state health departments and other anecdotal evidence [26,27,28,29,30]. The disparities apparent by race/ethnicity may be attributable to the legacy of institutional racism that influences the rates of chronic diseases and access to healthcare coverage [31]. Taken together, the convergence of these sociodemographic and structural factors may be essential elements to consider in understanding the burden of COVID-19 within the U.S.

Although COVID-19 data continue to emerge, limited research explores the spatial clustering of COVID-19 cases. To the best of our knowledge, only one study used geographically weighted regression models (i.e., examining spatial relationships at a variety of geographic scales that provide nonparametric estimates) to better elucidate geospatial patterns of COVID-19 incidence proportions within the continental U.S., concerning sociodemographic and environmental variables [32,33]. These results indicated that a combination of income indicators, healthcare professionals, and the percentage of Black females could explain the variability of disease incidence within the contiguous U.S. [32]. However, the prior study is limited in providing descriptive characteristics of the counties based on their clustering type. Therefore, their analyses suggest regions or states that have a higher disease burden.

There are also studies that have used complex modeling to examine some of the racial/ethnic disparities in COVID-19 rates in the United States. Specifically, researchers used structured compartmental models for seroprevalence data from the state of New York to examine immunity thresholds, final sizes, and COVID-19 risk across groups [34]. Their models suggest that the higher cumulative incidence for Hispanics and non-Hispanic Blacks compared to non-Hispanic Whites reflected the different racial/ethnic inequalities in both individual and community level socioeconomic status indicators [34]. Additionally, researchers have used machine learning methods to examine the role of racial residential segregation on COVID-19 infection and mortality [35]. Their models suggest that counties that are a standard deviation above the mean for racial residential segregation were more likely to have infection and mortality rates that were higher than other counties [35]. These studies suggest the inequitable burden of the COVID-19 pandemic on communities of color within the United States.

In contrast, spatial clustering analyses with Moran’s I can determine specific counties with higher or lower case rates relative to their surrounding counties. Additionally, spatial clustering analyses can identify sociodemographic characteristics that may influence COVID-19 incidence. Thus, the objectives of this study were to use spatial clustering analyses to determine whether COVID-19 incident proportions vary spatially in the U.S., how spatial clustering may change over time, and sociodemographic characteristics of counties within high COVID-19 incident proportion clusters in the U.S. We hypothesize that there would be higher COVID-19 clusters in areas with lower socioeconomic status and adverse community conditions.

## 2. Materials and Methods

### 2.1. Data and Measures

The number of COVID-19 cases by county was downloaded from U.S.A. Facts. Four distinct periods were created to better understand changes in spatial clusters by each period, which were defined as quartiles based on the cumulative cases as of 30 April 2021: 22 January–16 May 2020, 17 May–9 September 2020, 10 September–4 January 2021, and 5 January 2021–30 April 2021. The total number of persons residing in each county was gathered from 2019 Estimates, which was downloaded from the U.S. Census Bureau [36].

To standardize the number of COVID-19 cases across the U.S., the number of COVID-19 cases for each county was divided by the total population within each county and then multiplied by 100,000. The COVID-19 incident proportion is defined as the total number of positive cases divided by the total population within each county. The incident cases for each period were created by subtracting the case numbers on the first day of the study period from the number on the last day of the study period and multiplying each by 100,000 to obtain rates per 100,000 persons. An incident proportion was created for each county in the contiguous U.S., excluding Hawaii, Alaska, and U.S. territories [37]. Additional data on county-level racial/ethnic and age composition, socioeconomic factors, health outcomes, and health behaviors were downloaded from the 2020 Robert Wood Johnson Foundation’s County Health Indicator data (Table A1) [38]. The county level characteristics that were selected for this study have been used in previous studies [39,40,41,42].

### 2.2. Geographic Information System (G.I.S.) Process

2018 U.S. county cartographic boundary shapefiles were downloaded from the U.S. Census Bureau and uploaded into ArcGIS 10.5.1 (ESRI, Redlands, CA, USA). However, only counties in the contiguous U.S. were included in the analyses [40]. Using choropleth maps, the COVID-19 incident proportions during each of the periods were mapped in ArcGIS 10.5.1 across the contiguous U.S. counties and independent cities.

### 2.3. Statistical Analysis

Four distinct periods of the data were analyzed in a two-step process. Briefly, during the first step, Global Moran’s I was used to examine if COVID-19 incident proportions on the county level were spatially autocorrelated [43]. These values range from −1 to +1. If the Moran’s I value is positive, there is a clustering of COVID-19 incident proportions within the surrounding geographic area (i.e., counties). If the Moran’s I value is negative, the COVID-19 incident proportions are dispersed across the geographic area. Furthermore, inverse distance was applied to examine these spatial relationships, where nearby county COVID-19 incident proportions have a more significant impact on the computations for each county compared to counties that are further away, as previously carried out [40,44]. Z-scores refer to standard deviations from the mean; the more significant the standard deviation, the greater the results are from the mean or standard normal distribution [45]. These analyses identify statistically significant clusters of COVID-19 across the contiguous counties in the 48 U.S. states.

The second step of the data analysis involved using the Anselin Local Moran’s I to identify the specific counties within the contiguous U.S. with high and low COVID-19 incident proportions statistically different from nearby counties [46]. The results of this analysis provide a map of the spatial distribution of clustering to identify hot spots (counties with high COVID-19 incident proportions), cold spots (counties with low COVID-19 incident proportions), spatial outliers (counties with COVID-19 incident proportions that differ from nearby counties), and clusters that do not fall within any cluster type. This tool classifies each county into the groups mentioned above by using the COVID-19 prevalent/incident cases and creating a local Moran’s I value, a z-score, a p-value, and a classification identifier [46]. The final z-score and *p*-values for each county represent the likelihood of a statistically significant difference in each county’s COVID-19 incident proportions [46]. These results also allow for a basic comparison between the characteristics of the hot and cold spot clusters and their outliers. We then used a t-test to compare each cluster type to each unclustered county to test for a statistical difference in the county’s sociodemographic characteristics [47].

## 3. Results

### 3.1. Overall Distribution of COVID-19 Cases

**Figure 1:**
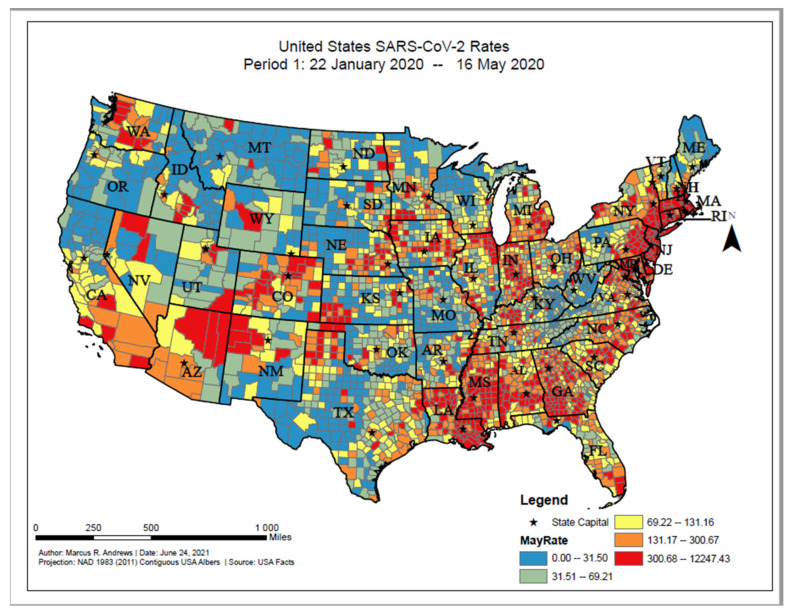
United States SARS-CoV-2 proportions, Period 1: 22 January 2020–16 May 2020.

The overall distribution of COVID-19 cases between the first reported COVID-19 case in the U.S. on 22 January 2020 and 16 May 2020 ranged from 0 to 12,247.43 cases per 100,000 persons across the U.S. counties (Figure 1). Based on the geographic distribution of COVID-19, the highest COVID-19 incident proportions were found among counties in the New England and mid-Atlantic areas (primarily Massachusetts, Rhode Island, Connecticut, New York, New Jersey, Maryland, and Pennsylvania), southern Louisiana, southeastern Michigan, western New Mexico, southern Alabama, southern Mississippi, southern Georgia, northern Arizona, and northwestern Washington. The value of Moran’s I for the contiguous areas of the U.S. was 0.25; since this value is positive, it indicates that the COVID-19 cases were spatially clustered on the county level. Since the z-score was 50.72, there was a less than 1% chance that this pattern could have occurred by chance.

**Figure 2:**
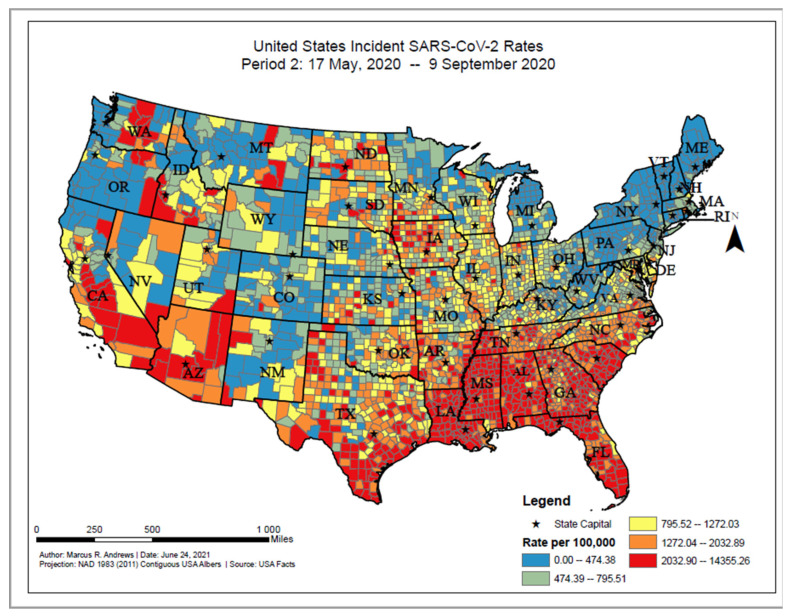
United States SARS-CoV-2 incident proportions, Period 2: 17 May 2020–9 September 2020.

The overall distribution of incident COVID-19 proportions ranged from 0 to 14,355.26 per 100,000 persons between 17 May 2020 and 9 September 2020. Based on the geographic distribution of COVID-19, higher incident cases could be found among counties widespread in the Southern states (e.g., South Carolina, Florida, and Mississippi), southern and eastern Arizona, and interior California (Figure 2). The value of Moran’s I for the contiguous areas of the U.S. was 0.47; since this value is positive, it indicates that the COVID-19 cases were spatially clustered on the county level. Since the z-score was 92.36, there was a less than 1% chance that this pattern could have occurred by chance.

**Figure 3:**
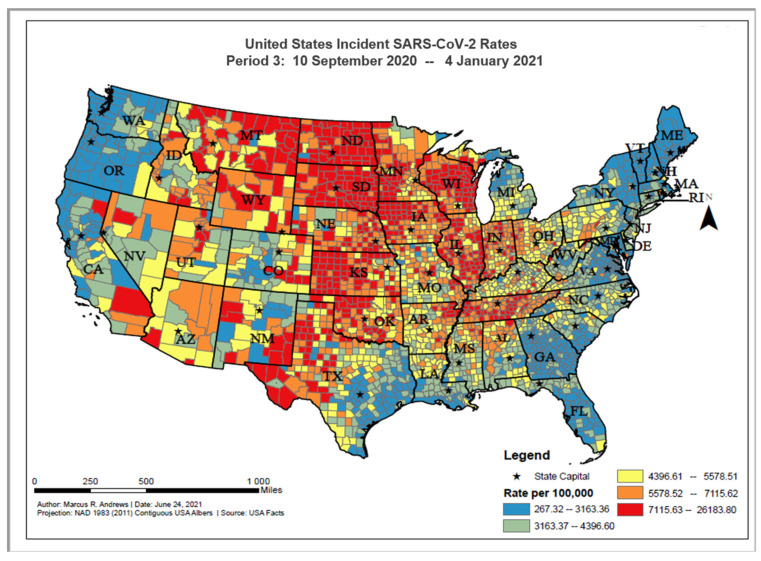
United States SARS-CoV-2 incident proportions, Period 3: 10 September 2020–4 January 2021.

The overall distribution of incident COVID-19 proportions ranged from 0 to 14,094.04 per 100,000 persons between 10 September 2020 and 4 January 2021. Based on the geographic distribution of COVID-19, higher incident cases can be found among counties in the Midwest (e.g., the Dakotas, Wisconsin, and Kansas), Tennessee, western Texas, and eastern New Mexico (Figure 3). The value of Moran’s I for the contiguous areas of the U.S. was 0.53; since this value is positive, it indicates that the COVID-19 cases were spatially clustered on the county level. Since the z-score was 105.12, there was a less than 1% chance that this pattern could have occurred by chance.

**Figure 4:**
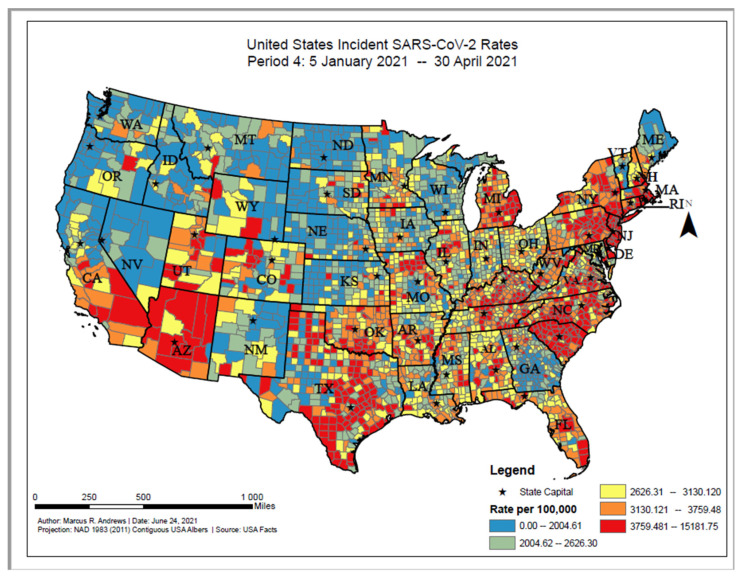
United States SARS-CoV-2 incident proportions, Period 4: 5 January 2021–30 April 2021.

The overall distribution of incident COVID-19 proportions ranged from 0 to 15,181.75 per 100,000 persons between 5 January 2021 and 30 April 2021. Based on the geographic distribution of COVID-19, higher incident cases were found among counties in the southwest (e.g., Arizona and interior California), interior Texas, eastern Michigan, and along the East Coast (e.g., Massachusetts, New Jersey, the Carolinas) (Figure 4). The value of Moran’s I for the contiguous areas of the U.S. was 0.35; since this value is positive, it indicates that the COVID-19 cases were spatially clustered on the county level. Since the z-score was 68.37, there was a less than 1% chance that this pattern could have occurred by chance.

### 3.2. Anselin’s Local Moran’s I

**Figure 5:**
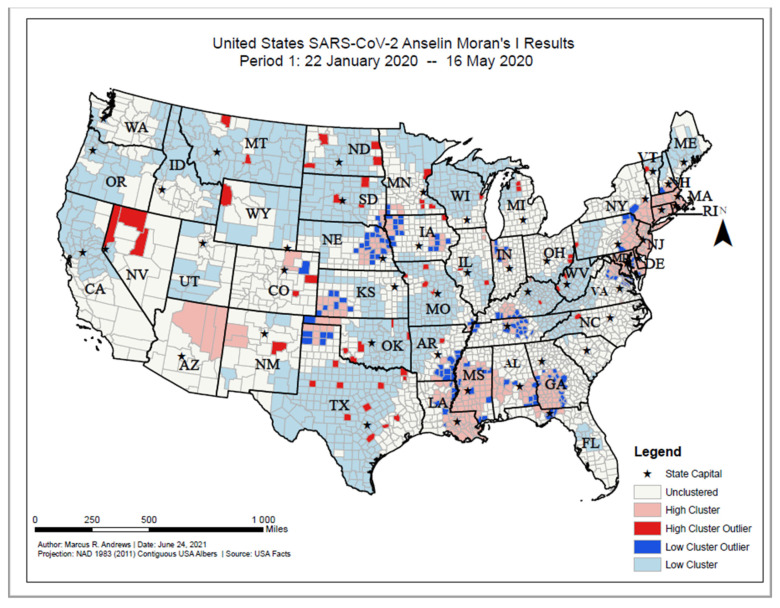
United States SARS-CoV-2 Anselin Moran’s I results, Period 1: 22 January 2020–16 May 2020.

The Anselin Local Moran’s I classified each county in the contiguous U.S. based on their similarities or differences. Based on the results of the Anselin Local Moran’s I, widespread clusters of high COVID-19 incident proportions, as of 16 May 2020, were statistically significant and located in the New England and Mid-Atlantic states (e.g., Massachusetts, New York, and Delaware), southwestern Georgia, Mississippi, southern Alabama, southeastern Louisiana, northern Texas, western Oklahoma, southwestern Kansas, and northern Arizona (Figure 5). High prevalence COVID-19 county outliers were primarily found in northern Nevada. Clusters of counties with low numbers of COVID-19 cases were significantly located in the Appalachian Mountains area, the midwestern states (including Wisconsin, Minnesota, North and South Dakota, Illinois, Nebraska, Iowa, and Montana) southern states (including Missouri, Arkansas, Kansas, Oklahoma, and Texas) and western states (e.g., Oregon, northern California, southern Utah, southeastern Idaho, eastern Wyoming). Low prevalence outliers were primarily concentrated in southeastern Nebraska, western Iowa, northern Texas, southeastern Arkansas, northern Mississippi, central Alabama, and central Georgia.

**Figure 6:**
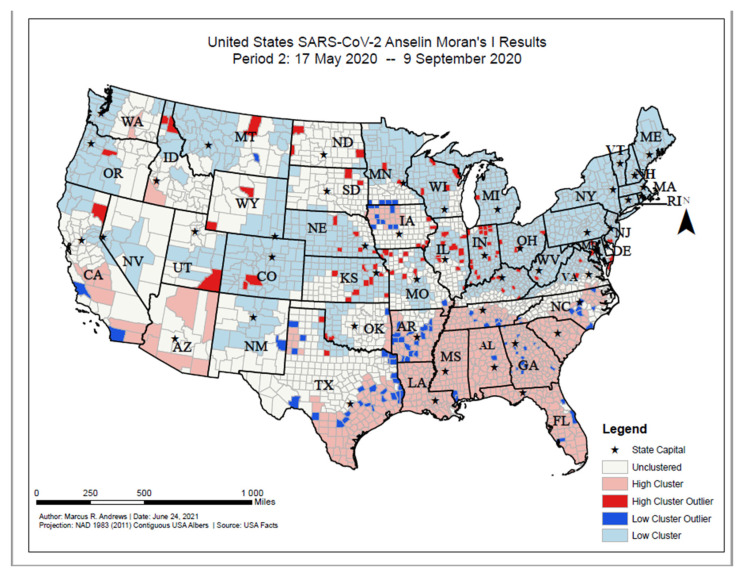
United States SARS-CoV-2 Anselin Moran’s I results, Period 2: 17 May 2020–9 September 2020.

As of 9 September 2020, high COVID-19 incident proportion clusters were primarily located in Southern states (Georgia, Alabama, Mississippi, Arkansas, southeastern Texas, and Louisiana), northwestern Iowa, Arizona, and interior California (Figure 6). Clusters with low COVID-19 incident cases were primarily located in New England and Mid-Atlantic states, midwestern states, and western states (e.g., Montana, Idaho, Utah, northern California, Oregon, and Washington). High incidence outlier counties were located across the United States and included counties in Indiana, Illinois, Wisconsin, Missouri, the Dakotas, Nebraska, and Kansas. Low incidence outlier counties were primarily located in North Carolina, Georgia, Florida, Arkansas, southeastern Texas, southern Minnesota, and northwestern Iowa.

**Figure 7:**
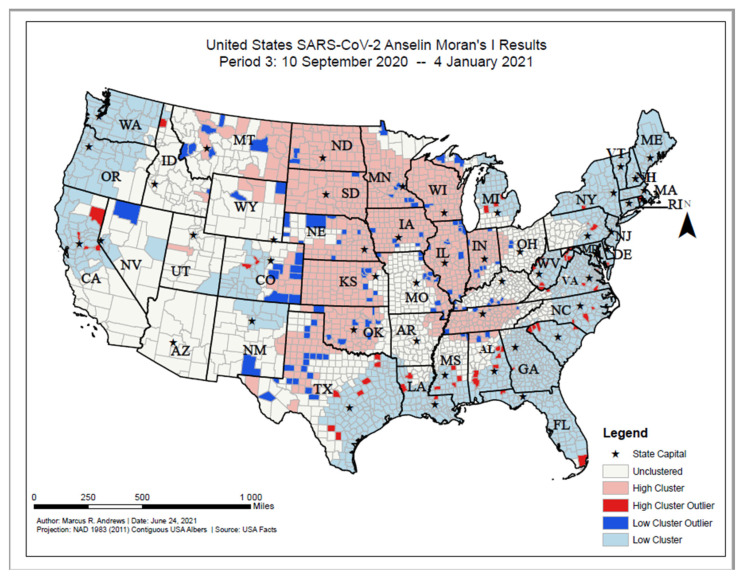
United States SARS-CoV-2 Anselin Moran’s I results, Period 3: 10 September 2020–4 January 2021.

As of 4 January 2021, high COVID-19 incident proportion clusters were primarily located in the central U.S. and included North and South Dakota, Minnesota, Wisconsin, Illinois, Indiana, Nebraska, Kansas, Oklahoma, Missouri, Montana, Wyoming, Arkansas, Tennessee, and Texas (Figure 7). Low incident clusters were located primarily along the coastal regions of the U.S., stretching from Maine to Florida westward through Georgia, Louisiana, and southeastern Texas. Low incident clusters were also located from Washington state to central California. High incident outlier counties were located primarily in Alabama, South Carolina, eastern Texas, and northern California. Low incident outlier counties were primarily located in the central U.S., stretching from the Dakotas to the north, Montana to the west, western Ohio to the east, and western Texas to the South.

**Figure 8:**
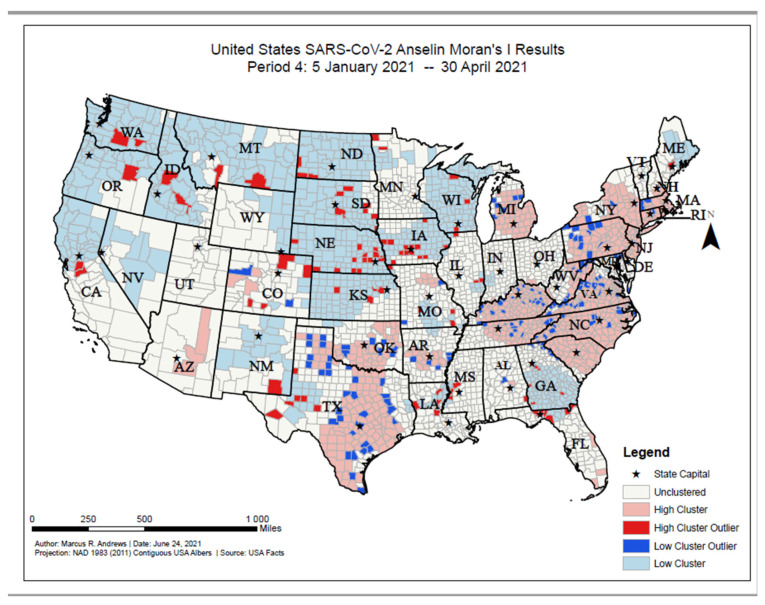
United States SARS-CoV-2 Anselin Moran’s I results, Period 4: 5 January 2021–30 April 2021.

As of 30 April 2021, high COVID-19 incident proportion clusters were primarily located along the eastern U.S, ranging from southern New Hampshire to South Carolina. High incident proportion clusters were also located in Kentucky, Michigan, northeastern Tennessee, Oklahoma, Texas, and eastern Arizona (Figure 8). Low incident proportion clusters were primarily located in the western U.S., including Washington, Oregon, northern California, Nevada, New Mexico, Montana, and Idaho. Low incident proportion clusters were also located in the Dakotas, Nebraska, Kansas, Iowa, southern Kansas, Wisconsin, Minnesota, and Georgia. High incident outlier counties were located primarily in Nebraska, Iowa, Washington, Idaho, Colorado, and Texas. Low incident outlier counties were located primarily in Texas, Oklahoma, Tennessee, the Carolinas, Virginia, and Pennsylvania.

### 3.3. Comparison of Clustering Characteristics

Overall, there are statistically significant differences in the spatial distribution of clusters of both high and low COVID-19 incident proportions across the four time points in this study (Table 1). As of 16 May 2020, high COVID-19 cluster counties were more likely than unclustered counties to have higher % female, % under 18, % Black, % Asian, Median Household Income, % Single Parent Households, % Food Insecure, % Unemployed, % Adults with Diabetes, % Fair or Poor Health, % Smokers, % Physically Inactive, and % Severe Housing Issues (Table 1). Low COVID-19 cluster counties were more likely than unclustered counties to have higher % Rural, % 65 and older, % White, High School Graduation Rate, and % Smokers. High COVID-19 cluster outlier counties were more likely than unclustered counties to have higher % Native American, % Hispanic, and % Uninsured. Low COVID-19 cluster outlier counties were more likely than unclustered counties to have higher % Rural, % Black, % Food Insecure, % Fair or Poor Health, and % Adults with Obesity.

As of 9 September 2020, high COVID-19 cluster counties were more likely than unclustered counties to have higher % Female, % Black, % Single Parent Household, % Severe Housing Cost Burden, % Food Insecure, % Uninsured, % Unemployed, and % Adults with Diabetes, (Table 2). Low COVID-19 cluster counties were more likely than unclustered counties to have higher % 65 and older, % White, Black/White Segregation, % Some College, % Homeowners, and % Smokers. High COVID-19 cluster outlier counties were more likely than unclustered counties to have higher % Female, % Black, % Smokers, and % Adults with Obesity. Low COVID-19 cluster outlier counties were more likely than unclustered counties to have higher % Rural and % Female.

As of 4 January 2021, high COVID-19 cluster counties were more likely than unclustered counties to have higher % under 18, % White, % Native American, Black/White Segregation, Median Household Income, High School Graduation Rate, % Homeowners, and Life Expectancy (Table 3). Low COVID-19 cluster counties were more likely than unclustered counties to have higher % Black, % Asian, % Single Parent Household, and % Severe Housing Cost Burden. High COVID-19 cluster outlier counties were more likely than unclustered counties to have higher % Single Parent Households, % Severe Housing Issues, and % Adults with Diabetes, to name a few. Low COVID-19 cluster outlier counties were more likely than unclustered counties to have higher % Rural, % 65 and older, % Asian, High School Graduation Rate, % Some College, and Food Environment Index.

As of 30 April 2021, high COVID-19 cluster counties were more likely than unclustered counties to have a higher % Asian (Table 4). High COVID-19 cluster outlier counties were more likely than unclustered counties to have a higher % Asian. Low COVID-19 cluster outlier counties were more likely than unclustered counties to have higher Black/White Segregation and % Severe Housing Cost Burden.

## 4. Discussion

Our results show that the U.S. has significant disparities in the geographic distribution and clustering of COVID-19 cases that vary based on the time period. In the initial months of the COVID-19 pandemic, widespread areas with the highest COVID-19 incident proportions were in counties within New England, along the East Coast, and areas throughout the South. However, as the pandemic continued, high incident clusters moved from widespread areas in the Midwest back to counties along the East Coast and South. Furthermore, incidence proportions also revealed a similar trend. As the pandemic has continued, rural counties within the United States have become increasingly more prevalent in the high COVID-19 cluster areas. Racial and ethnic minorities have tended to be classified in the high COVID-19 cluster areas at one point during the pandemic; however, counties with higher percentages of Blacks, Hispanics, and Asians tended to remain in either high COVID-19 clusters or high COVID-19 outliers. Our results suggest that counties with higher percentages of Whites had remained in the low COVID-19 cluster counties until Period 3: 19 September–4 January, when counties with higher percentages of Whites became classified as high COVID-19 cluster counties. Furthermore, in the initial periods of the pandemic, counties that were considered in the low cluster areas were more likely to have higher Black/White segregation. Comparing trends between county characteristics suggests more significant differences in county characteristics during the initial periods of the pandemic, whereas more recent data suggests that there are not as many significant differences between county demographics.

Our findings around the inequitable adverse county-level exposures associated with higher clusters of COVID-19 cases contribute to the emerging literature around the inequitable distribution of COVID-19 in the U.S.A recent spatial analysis of COVID-19 suggested that several factors, including sociodemographic characteristics and comorbidities, would be associated with areas with an increased COVID-19 burden [7]. Additionally, as more research has emerged around the COVID-19 pandemic, analyses have suggested that more vulnerable populations may be at an increased risk of COVID-19 infection. Specifically, counties that have a higher minority population and lower English language proficiency may be more vulnerable than counties that are predominately White and native English speakers [25]. Furthermore, these relationships have also been suggested by machine learning and other modeling techniques, which have shown a disproportionate burden of COVID-19 infection and mortality among communities of color within the United States [34,35]. Again, structural inequalities likely contribute to these disparities that are apparent in the existing literature and our study. Like prior findings around sociodemographic characteristics and health status, we demonstrated that the counties that fell into statistically significant higher prevalence or incidence cluster areas had a lower percentage of Whites and an increasing percentage of households with overcrowding and cost concerns.

The outcomes of our Anselin Local Moran’s I analyses suggest that inadequate housing, under/unemployment, preexisting adverse health conditions (i.e., diabetes and obesity), health behaviors (i.e., physical inactivity and smoking), and household characteristics, in addition to segregation along racial lines, have created additive effects of social determinants of health that may help to explain some the inequitable distribution and unequal risk of COVID-19 among minority communities within the contiguous U.S. These conditions culminate in chronic stress, which may impair the immune system, potentially increasing susceptibility to COVID-19 and adverse health complications [48,49,50,51].

The results from this study can be used to help inform public policy for mitigating COVID-19 risk. The cluster demographics results can be used to identify emergent cases and clusters of COVID-19 further in order to develop a more robust infrastructure to monitor COVID-19 infections [52]. Our results suggest that, until the fourth period of analysis in this study, counties with higher percentages of Blacks, Hispanics, and Asians were more likely to be classified within the high cluster areas. Similarly, counties with higher percentages of Whites remained in the low cluster categories until the third analysis period for this study, when counties with higher percentages of Whites were classified in the high cluster categories. Our results are similar to a recent study examining the geographical variations of COVID-19 cases and demographic characteristics [53]. Like our results, this study found a significant correlation between county percentages of Blacks and COVID-19 cases and deaths—however, these results did not remain among Hispanic populations.

Furthermore, counties with higher levels of Whites were negatively correlated with COVID-19 cases and deaths. Our findings around the potential racial burden of COVID-19 are also supported by a recent study by Mahajan and Larkins-Pettigrew. Their study examined COVID-19 and race by county [54]. Like our findings, their results suggest a positive correlation between county percentages of both Blacks and Asians and COVID-19 cases. However, both our study and theirs suggest that counties with a higher percentage of Whites had lower COVID-19 cases and deaths.

This investigation has several strengths and limitations. The strengths of this study include testing for spatial autocorrelations using Moran’s I and Anselin Local Moran’s I, which have not been conducted to test spatial clustering of COVID-19 incident proportions across U.S. counties. Furthermore, to the best of our knowledge, this is the first study that explores the sociodemographic characteristics of the different clusters of COVID-19 within the continental U.S. Limitations include the delayed onset of COVID-19, which may underrepresent the published data used for the analysis of the study. The number of cases included in these analyses is those tested for COVID-19; this number could be underreported, as some people may be asymptomatic or may opt out of COVID-19 testing. Additionally, the county characteristics reflect patterns at the county level and not the characteristics of those who tested positive for the virus. Furthermore, our analyses are unable to account for the confounding of local policies with public health interventions that may relate to differences in case numbers within these counties.

Additionally, using counties as the geographic scale may mask the actual dispersion of COVID-19 incident proportions within counties. As mentioned, census tracts or census block groups may be at a more granular scale. However, all of these geographic scales may be subject to the modifiable areal unit problem (i.e., geographical boundaries may change over time, altering the comparison of the results across multiple years [40,55]. Furthermore, smaller administrative boundary areas, such as counties and/or zip codes, may be the most granular level on which these data can be represented due to privacy concerns. Future research could potentially utilize granular data at the census tract level, for example, while protecting the privacy of less populated census tracts or block groups.

## 5. Conclusions

Despite the dearth of research using geographic information systems to examine COVID-19, we sought to examine the spatial distribution of COVID-19 within the continental U.S. using geospatial statistical analyses. The results of our analyses suggest that several sociodemographic variables are correlated with higher county-level proportions of COVID-19. The results of this study may be helpful for health policy decision-makers in their attempts to provide vital public health resources and dismantle some of the structural barriers faced by some residents within high COVID-19 prevalence or incidence clusters. These types of spatial clustering maps can allow state and county leaders to strategically mitigate an increase in incident proportions in their jurisdictions based on public health evidence. By identifying spatial clusters, state and local-level government leaders can more effectively monitor their jurisdictions in a public health-informed manner to prevent an uncontrollable spread of COVID-19. Ultimately, these data can be used to prioritize and reallocate vital public health, treatment, and testing equipment to the most impacted areas. It is also important to note that this may not be the last pandemic. The strategies used to mitigate this pandemic may help to prevent or address future pandemics.

## Figures and Tables

**Table 1:** County characteristics based on Anselin Moran’s I results for Time Point 1.

	Time Point 1: Rates from 22 January–16 May 2020 Results				
	Unclustered (*n* = 1531)	High COVID-19 Rate Clusters (*n* = 349)	Low COVID-19 Rate Clusters (*n* = 1021)	High COVID-19 Rate Outlier (*n* = 57)	Low COVID-19 Rate Outlier (*n* = 150)
Range					
Characteristics (S.D.)					
Cases per 100,000 people	228.13 (534.16)	855.73 (788.15) ***	47.75 (45.53) ***	452.50 (216.06) **	133.67 (63.33) *
% Rural	56.49% (31.12%)	45.96% (33.57%) ***	64.72% (29.76%) ***	54.39% (30.74%)	68.20% (28.72%) ***
% Female	49.94% (2.17%)	50.64% (2.43%) ***	49.72% (2.08%) *	48.56% (4.02%) ***	50.01% (2.33%)
% of Age Group (S.D.)					
% under 18	22.00% (3.26%)	22.69% (3.25%) **	21.90% (3.76%)	22.19% (3.16%)	22.31% (2.86%)
% 65 and older	19.05% (4.50%)	17.21% (3.50%) ***	20.52% (5.02%) ***	18.47% (4.36%)	19.15% (4.27%)
Race/Ethnicity (S.D.)					
% White	76.66% (18.36%)	61.02% (20.60%) ***	81.00% (19.56%) ***	75.19% (19.03%)	77.33% (17.67%)
% Black	9.94% (13.81%)	23.34% (21.92%) ***	2.43% (3.62%) ***	4.74% (5.37%) *	14.07% (17.25%) **
% Native American	1.27% (3.45%)	1.64% (7.58%)	3.40% (9.67%) ***	3.42% (7.76%) ***	0.50% (0.68%) *
% Asian	1.53% (2.60%)	2.62% (3.91%) ***	1.06% (1.73%) ***	1.32% (1.65%)	1.01% (1.86%)
% Hispanic	9.27% (12.47%)	10.17% (11.57%)	10.50% (16.91%) *	14.00% (15.82%) *	5.66% (5.83%) *
% Native Hawaiian/Pacific Islander	0.02% (0.17%)	0.01% (0.13%)	0.04% (0.24%) *	0.07% (0.26%) *	0.01% (0.08%)
Black/White Segregation	44.61 (16.55)	42.53 (15.96) *	49.97 (15.02) ***	47.07 (15.67)	35.11 (16.33) ***
Median Household Income	53,674.39 (13,082.72)	56,927.61 (21,725.10) *	49,665.37 (10,315.00) ***	52,178.53 (12,946.71)	52,934.70 (14,757.76)
Community Characteristics (S.D.)					
High School Graduation Rate	88.76% (6.51%)	86.59% (7.33%) ***	89.64% (7.58%) *	89.02% (6.34%)	89.80% (7.91%)
% Some College	58.43% (11.60%)	57.54% (13.10%)	57.25% (11.33%) **	55.63% (13.40%)	58.46% (13.50%)
% Homeowners	71.52% (7.83%)	68.92% (9.86%) ***	72.54% (7.50%) **	70.21% (8.16%)	71.61% (9.31%)
% Single Parent Household	32.41% (9.92%)	38.55% (14.45%) ***	30.28% (9.22%) ***	30.89% (7.38%)	31.69% (12.10%)
% Severe Housing Issues	13.69% (3.86%)	15.94% (4.67%) ***	13.27% (4.12%) *	12.35% (3.83%) *	12.65% (3.99%) **
% Severe Housing Cost Burden	11.28% (3.44%)	13.45% (4.12%) ***	10.64% (3.26%) ***	10.12% (3.41%) *	10.95% (3.95%)
% Food Insecure	13.08% (3.57%)	14.53% (6.33%) ***	13.12% (3.15%)	12.65% (3.63%)	13.81% (5.11%) *
Food Environment Index	7.56 (0.99)	7.26 (1.61) ***	7.39 (1.063) ***	7.55 (1.12)	7.43 (1.43)
% Uninsured	12.93% (5.80%)	13.60% (6.41%)	14.34% (6.67%) ***	15.16% (7.50%) **	13.05% (4.82%)
% Unemployed	4.02% (1.30%)	4.47% (1.60%) ***	4.15% (1.49%) *	3.84% (1.28%)	3.83% (1.49%)
County Level Health Related factors (S.D.)					
Life Expectancy	77.64 (2.91)	77.30 (3.35)	77.25 (3.03) **	77.87 (3.44)	77.13 (2.86) *
% Adults with Diabetes	12.14% (4.01%)	12.68% (4.46%) *	12.04% (3.94%)	12.32% (4.63%)	12.81% (4.22%)
% Fair or Poor Health	17.48% (4.14%)	19.63% (5.99%) ***	18.00% (4.81%) **	17.75% (4.63%)	18.48% (5.70%) **
% Smokers	17.22% (3.10%)	17.81% (3.98%) **	17.56% (4.01%) *	17.23% (3.30%	18.13% (3.76%) **
% Adults with Obesity	32.90% (5.41%)	33.47% (6.31%)	32.43% (4.98%) *	32.77% (6.77%)	34.83% (5.34%) ***
% Physically Inactive	27.30% (5.76%)	28.16% (6.22%) *	27.26% (5.18%)	26.51% (6.27%)	29.47% (6.36%) ***

Note: T-test were used to compare values for each “Unclustered Counties” to each remaining group. *p*-value: *** < 0.001; ** <0.01; * < 0.05.

**Table 2:** County characteristics based on Anselin Moran’s I results for Time Point 2.

	Time Point 2: Rates from 17 May–9 September 2020 Results Incident Case Cluster				
	Unclustered (*n* = 946)	High COVID-19 Rate Clusters (*n* = 717)	Low COVID-19 Rate Clusters (*n* = 1238)	High COVID-19 Rate Outlier (*n* = 109)	Low COVID-19 Rate Outlier (*n* = 114)
Range					
Characteristics (SD)					
Cases per 100,000 people	1264.75 (793.22)	2715.26 (1311.74) ***	565.76 (310.83) ***	1757.13 (429.08) ***	1039.71 (232.85) *
% Rural	58.94% (33.65%)	56.80% (28.95%)	59.53% (30.87%)	47.70% (33.61%) **	66.87% (27.97%) *
% Female	49.73% (2.09%)	50.23% (2.91%) ***	49.84% (1.96%)	50.16% (2.03%) *	50.39% (1.43%) *
% of Age Group (S.D.)					
% under 18	22.90% (3.86%)	22.53% (3.11%) *	21.15% (3.03%) ***	22.44% (3.40%)	21.70% (2.87%) *
% 65 and older	19.21% (5.00%)	18.07% (4.40%) ***	20.15% (4.36%) ***	17.78% (4.21%) *	20.70% (5.17%) *
Race/Ethnicity (S.D.)					
% White	75.90% (19.50%)	60.87% (20.52%) ***	85.59% (13.49%) ***	79.41% (16.03%)	73.41% (18.16%)
% Black	4.94% (8.88%)	24.12% (19.60%) ***	3.44% (5.60%) ***	7.08% (10.81%) *	12.42% (14.45%) ***
% Native American	3.40% (9.56%)	1.15% (4.14%) ***	1.57% (5.36%) ***	1.53% (5.11%) *	1.21% (2.53%) *
% Asian	1.65% (3.20%)	1.20% (1.53%) **	1.46% (2.47%)	1.81% (2.31%)	1.30% (2.18%)
% Hispanic	12.65% (15.43%)	11.50% (17.81%)	6.42% (8.91%) ***	8.54% (8.26%) ***	10.47% (13.51%)
% Native Hawaiian/Pacific Islander	0.05% (0.25%)	0.02% (0.19%) *	0.02% (0.14%) **	0.04% (0.19%)	0.01% (0.10%)
Black/White Segregation	46.0475 (15.36)	37.0831 (14.38) ***	52.8096 (15.10) ***	49.4997 (16.15) *	34.6357 (15.19) ***
Median Household Income	54,365.72 (13,835.57)	46,256.74 (11,294.04) ***	55,251.09 (14,359.52)	52,879.94 (9623.77)	50,052.32 (12,506.54) **
Community Characteristics (S.D.)					
High School Graduation Rate	89.60% (7.37%)	86.94% (6.95%) ***	89.29% (6.84%)	89.46% (6.50%)	89.88% (6.81%)
% Some College	59.32% (11.78%)	51.41% (11.15%) ***	60.66% (10.71%) *	59.80% (11.91%)	54.47% (12.31%) ***
% Homeowners	71.48% (8.08%)	69.50% (8.34%) ***	72.87% (7.60%) ***	67.97% (9.84%) ***	74.32% (6.73%) **
% Single Parent Household	29.56% (9.77%)	40.28% (11.66%) ***	29.87% (8.40%) ***	31.87% (8.97%)*	32.81% (9.67%) **
% Severe Housing Issues	13.25% (4.44%)	15.21% (3.76%)	13.27% (3.94%)	13.74% (4.00%)	13.38% (3.67%)
% Severe Housing Cost Burden	10.53% (3.59%)	12.49% (3.55%) ***	11.17% (3.47%) ***	11.21% (3.60%)	10.96% (3.38%)
% Food Insecure	12.49% (3.61%)	16.17% (4.72%) ***	12.11% (2.73%) *	12.94% (2.95%)	14.92% (4.42%) ***
Food Environment Index	7.512 (1.17)	6.910 (1.20) ***	7.759 (0.92) ***	7.600 (0.90)	7.148 (1.24) **
% Uninsured	14.65% (6.66%)	17.53% (5.41%) ***	10.21% (4.25%) ***	12.13% (4.91%) ***	16.37% (6.49%) *
% Unemployed	3.70% (1.30%)	4.59% (1.54%) ***	4.15% (1.35%) ***	3.78% (1.34%)	4.10% (1.24%) *
County Level Health Related factors (S.D.)					
Life Expectancy	77.8875 (3.12)	75.94 (2.56) ***	78.05 (2.94)	77.56 (2.52)	76.97 (2.61) *
% Adults with Diabetes	11.46% (3.93%)	14.35% (4.22%) ***	11.43% (3.49%)	11.83% (3.59%)	13.94% (5.25%) ***
% Fair or Poor Health	17.04% (4.23%)	21.90% (4.75%) ***	16.32% (3.84%) ***	17.39% (3.61%)	19.07% (4.11%) ***
% Smokers	16.48% (3.67%)	19.18% (3.19%) ***	17.15% (3.43%) ***	17.94% (3.08%) ***	17.22% (2.87%) *
% Adults with Obesity	32.06% (5.00%)	35.26% (5.59%) ***	32.02% (5.27%)	33.80% (4.67%) **	33.96% (5.75%) **
% Physically Inactive	26.55% (5.11%)	30.85% (5.72%) ***	26.08% (5.31%) *	26.84% (4.96%)	29.88% (5.75%) ***

Note: T-test was used to compare values for each “Unclustered Counties” to each remaining group. *p*-value: *** < 0.001; ** <0.01; * < 0.05.

**Table 3:** County characteristics based on Anselin Moran’s I results for Time Point 3.

	Time Point 3: Rates from 10 September–4 January 2021 Results Incident Case Cluster				
	Unclustered (*n* = 895)	High COVID-19 Rate clusters (*n* = 921)	Low COVID-19 Rate clusters (*n* = 1103)	High COVID-19 Rate Outlier (*n* = 65)	Low COVID-19 Rate Outlier (*n* = 124)
Range					
Characteristics (S.D.)					
Cases per 100,000 people	5327.78 (1605.30)	7804.44 (2106.94) ***	3088.61 (1058.54) ***	6069.39 (1060.79) **	4472.66 (785.93) ***
% Rural	59.33% (30.31%)	64.02% (29.78%) **	52.27% (32.03%) ***	57.49% (31.81%)	68.43% (35.31%) **
% Female	49.87% (2.24%)	49.68% (1.90%)	50.29% (2.34%) ***	48.97% (3.75%) *	49.40% (2.38%) *
% of Age Group (S.D.)					
% under 18	22.35% (3.39%)	23.02% (3.24%) ***	21.10% (3.27%) ***	20.45% (3.20%) ***	22.39% (3.87%)
% 65 and older	19.25% (4.59%)	19.54% (4.10%)	19.21% (5.16%) ***	18.91% (3.38%) ***	19.35% (5.30%) ***
Race/Ethnicity (S.D.)					
% White	78.53% (20.56%)	83.71% (15.89%) ***	68.27% (19.67%) ***	70.66% (19.79%) *	80.56% (16.50%)
% Black	6.60% (13.39%)	3.09% (5.79%) ***	16.22% (17.20%) ***	15.18% (15.51%) ***	4.74% (8.39%)
% Native American	2.01% (6.01%)	3.20% (10.24%) *	1.11% (2.62%) ***	1.43% (3.53%)	1.54% (2.77%)
% Asian	1.12% (1.75%)	0.98% (1.26%)	2.16% (3.54%) ***	1.34% (2.22%)	1.55% (2.75%) *
% Hispanic	10.34% (16.37%)	7.63% (11.31%) ***	10.83% (13.54%)	10.06% (14.58%)	10.00% (13.02%)
% Native Hawaiian/Pacific Islander	0.04% (0.23%)	0.02% (0.15%) *	0.03% (0.18%)	0.05% (0.21%)	0.02% (0.13%)
Black/White Segregation	48.72 (15.51)	50.81 (14.54) *	(40.79) (16.61) ***	40.34 (16.82) ***	45.22 (14.10)*
Median Household Income	49,097.69 (12,079.55)	53,713.01 (10,408.62) ***	54,742.55 (16,830.41) ***	48,150.68 (12,133.84)	54,379.05 (13,688.57) ***
Community Characteristics (S.D.)					
High School Graduation Rate	89.54% (7.09%)	90.34% (6.31%) *	86.85% (7.34%) ***	89.29% (6.05%)	91.00% (6.44%) *
% Some College	55.76% (11.18%)	61.00% (11.12%) ***	56.97% (12.09%) *	52.63% (11.88%) *	61.19% (13.44%) ***
% Homeowners	71.26% (7.32%)	73.25% (6.75%) ***	70.36% (9.23%) *	70.09% (9.14%)	72.23% (9.66%)
% Single Parent Household	31.85% (10.52%)	28.96% (9.61%) ***	35.87% (10.63%) ***	35.88% (8.23%) *	27.69% (9.88%) ***
% Severe Housing Issues	13.87% (3.84%)	11.35% (3.40%) ***	15.67% (3.85%) ***	14.98% (4.63%) *	12.49% (3.90%) **
% Severe Housing Cost Burden	11.06% (3.21%)	9.24% (2.62%) ***	13.20% (3.56%) ***	12.52% (4.08%) **	10.12% (3.53%) *
% Food Insecure	14.33% (3.73%)	11.46% (3.25%) ***	13.97% (4.25%) *	14.68% (3.68%)	12.42% (3.34%) ***
Food Environment Index	7.20 (1.14)	7.76 (1.09) ***	7.44 (1.09) ***	7.12 (1.15)	7.54 (1.11) *
% Uninsured	13.84% (6.11%)	12.13% (6.14%) ***	14.32% (6.08%)	14.25% (6.54%)	13.95% (6.47%)
% Unemployed	4.38% (1.56%)	3.47% (1.14%) ***	4.42% (1.32%)	4.58% (1.46%)	3.56% (1.23%) ***
County Level Health Related factors (S.D.)					
Life Expectancy	76.73 (2.80)	77.84 (2.97) ***	77.69 (3.00) ***	76.73 (4.33)	78.05 (3.17)* **
% Adults with Diabetes	12.43% (4.03%)	11.55% (3.65%) ***	12.63% (4.23%)	13.88% (5.20%) *	10.85% (4.20%) ***
% Fair or Poor Health	19.34% (4.82%)	16.25% (4.32%) ***	18.24% (4.60%) ***	20.03% (4.56%)	16.81% (4.20%) ***
% Smokers	18.32% (3.71%)	17.13% (3.66%) ***	17.01% (3.24%) ***	18.52% (3.65%)	16.74% (3.48%) ***
% Adults with Obesity	32.91% (5.39%)	33.27% (4.36%)	32.76% (6.11%)	33.54% (7.11%)	31.09% (5.07%) **
% Physically Inactive	28.19% (5.70%)	27.34% (4.80%) **	27.05% (6.23%) ***	28.63% (6.27%)	26.48% (5.97%) *

Note: T-test was used to compare values for each “Unclustered Counties” to each remaining group. *p*-value: *** < 0.001; ** < 0.01; * < 0.05.

**Table 4:** County characteristics based on Anselin Moran’s I results for Time Point 4.

	Time Point 4: Rates from 4 January–30 April 2021 Results Incident Case Cluster				
	Unclustered (*n* = 858)	High COVID-19 Rate Clusters (*n* = 742)	Low COVID-19 Rate Clusters (*n* = 1302)	High COVID-19 Rate Outlier (*n* = 92)	Low COVID-19 Rate Outlier (*n* = 114)
Range					
Characteristics (S.D.)					
Cases per 100,000 people	1947.35 (958.83)	3792.37 (1663.46) ***	906.91 (432.47) ***	2594.58 (781.70) ***	1558.95 (376.87) ***
% Rural	59.44% (31.32%)	59.23% (31.76%)	58.07% (30.97%)	55.89% (33.74%)	54.75% (33.46%)
% Female	49.91% (2.31%)	49.86% (2.32%)	49.99% (2.12%)	49.91% (2.92%)	49.67% (2.10%)
% of Age Group (S.D.)					
% under 18	22.11% (3.46%)	21.99% (3.24%)	22.06% (3.48%)	22.11% (3.47%)	22.21% (3.60%)
% 65 and older	19.39% (4.73%)	19.25% (4.72%)	19.38% (4.62%)	18.76% (4.25%)	19.02% (4.91%)
Race/Ethnicity (S.D.)					
% White	76.44% (19.79%)	75.46% (19.80%)	77.04% (19.63%)	73.63% (22.27%)	75.47% (21.15%)
% Black	9.29% (14.49%)	9.58% (14.90%)	8.66% (13.99%)	9.76% (15.02%)	8.51% (13.44%)
% Native American	1.91% (6.11%)	1.91% (5.99%)	2.14% (7.64%)	1.76% (3.69%)	2.13% (6.41%)
% Asian	1.28% (1.93%)	1.59% (2.67%) *	1.43% (2.54%)	2.23% (4.89%) **	1.85% (2.80%) *
% Hispanic	9.61% (14.47%)	10.07% (13.57%)	9.35% (13.37%)	11.10% (16.51%)	10.51% (15.10%)
% Native Hawaiian/Pacific Islander	0.04% (0.21%)	0.01% (0.11%) ***	0.03% (0.21%) ***	0.03% (0.18%) ***	0.04% (0.25%) ***
Black/White Segregation	45.07 (16.57)	43.80 (16.42)	45.81 (16.35)	44.24 (16.12)	48.73 (16.01) *
Median Household Income	52,846.36 (13,847.94)	53,031.23 (14,540.18)	52,124.25 (13,060.77)	54,360.28 (17,456.90)	53,576.16 (13,973.94)
Community Characteristics (S.D.)					
High School Graduation Rate	88.64% (7.32%)	89.13% (6.65%)	88.81% (7.24%)	88.95% (6.84%)	89.05% (6.57%)
% Some College	58.15% (11.62%)	57.65% (12.41%)	57.85% (11.59%)	58.14% (12.99%)	57.87% (11.55%)
% Homeowners	71.92% (8.44%)	71.46% (7.91%)	71.55% (7.90%)	70.79% (8.43%)	69.77% (9.16%) *
% Single Parent Household	32.56% (10.93%)	32.45% (10.82%)	32.22% (10.43%)	32.53% (11.38%)	31.16% (9.49%)
% Severe Housing Issues	13.60% (4.04%)	13.74% (4.03%)	13.73% (4.17%)	13.99% (4.80%)	14.42% (4.52%) *
% Severe Housing Cost Burden	11.23% (3.68%)	11.29% (3.59%)	11.19% (3.46%)	11.77% (4.36%)	12.11% (3.79%) *
% Food Insecure	13.32% (3.96%)	13.31% (4.10%)	13.26% (3.94%)	13.03% (4.23%)	13.22% (3.32%)
Food Environment Index	7.467 (1.06)	7.442 (1.19)	7.465 (1.14)	7.489 (1.21)	7.517 (0.97)
% Uninsured	13.53% (6.26%)	13.70% (6.17%)	13.37% (6.08%)	13.50% (7.29%)	13.89% (6.28%)
% Unemployed	4.06% (1.39%)	4.16% (1.47%)	4.09% (1.40%)	4.17% (1.50%)	4.05% (1.36%)
County Level Health Related factors (S.D.)					
Life Expectancy	77.40 (3.10)	77.46 (2.96)	77.42 (3.01)	77.87 (3.12)	77.84 (2.62)
% Adults with Diabetes	12.28% (4.24%)	12.09% (4.06%)	12.24% (4.02%)	12.35% (3.80%)	11.85% (3.61%)
% Fair or Poor Health	17.81% (4.76%)	18.14% (4.73%)	17.96% (4.73%)	17.74% (5.06%)	17.68% (4.39%)
% Smokers	17.36% (3.58%)	17.51% (3.54%)	17.53% (3.60%)	16.93% (3.59%)	17.06% (3.28%)
% Adults with Obesity	32.91% (5.61%)	33.03% (5.44%)	32.90% (5.34%)	33.16% (5.34%)	31.79% (5.07%) *
% Physically Inactive	27.55% (5.67%)	27.62% (5.79%)	27.49% (5.74%)	26.47% (5.62%)	26.62% (4.71%)

Note: T-test was used to compare values for each “Unclustered Counties” to each remaining group. *p*-value: *** < 0.001; ** < 0.01; * < 0.05.

## Data Availability

Data for these analyses are available at: https://usafacts.org/visualizations/coronavirus-covid-19-spread-map/ (accessed on 20 November 2020).

## Appendix A

**Table A1:** Sources for county-level characteristics.

Source	Variable	Definition
Behavioral Risk Factor Surveillance System	Poor or Fair Health	Percentage of adults reporting fair or poor health (age-adjusted).
	Adult smoking	Percentage of adults who are current smokers.
United States Diabetes Surveillance System	Adult obesity	Percentage of the adult population (age 20 and older) that reports a body mass index (BMI) greater than or equal to 30 kg/m^2^.
	Physical inactivity	Percentage of adults age 20 and over reporting no leisure-time physical activity.
	Diabetes prevalence	Percentage of adults aged 20 and above with diagnosed diabetes.
US Department of Agriculture Food Environment Atlas, Map the Meal Gap from Feeding America	Food environment index	Index of factors that contribute to a healthy food environment, from 0 (worst) to 10 (best).
State-specific sources and EDFacts (U.S. Department of Education)	High school graduation	Percentage of ninth-grade cohort that graduates in four years.
Bureau of Labor Statistics	Unemployment	Percentage of population ages 16 and older unemployed but seeking work.
American Community Survey, 5-year estimates	Residential segregation—Black/White	Index of dissimilarity where higher values indicate greater residential segregation between Black and White county residents.
	Homeownership	Percentage of occupied housing units that are owned.
	Severe housing cost burden	Percentage of households that spend 50% or more of their household income on housing.
	Some college	Percentage of adults ages 25–44 with some post-secondary education.
	Children in single-parent households	Percentage of children that live in a household headed by single parent.
Comprehensive Housing Affordability Strategy (CHAS) data	Severe housing problems	Percentage of households with at least one of four housing problems: overcrowding, high housing costs, lack of kitchen facilities, or lack of plumbing facilities.
Map the Meal Gap	Food insecurity	Percentage of population who lack adequate access to food.
National Center for Health Statistics—Mortality Files	Life expectancy	Average number of years a person can expect to live.
Small Area Health Insurance Estimates	Uninsured adults	Percentage of population under age 65 without health insurance.
	Median household income	The income where half of households in a county earn more and half of households earn less.
Census Population Estimates	Race/Ethnicity Data	Percentage of population that falls within each racial/ethnic category
	% Females	Percentage of population that is female.
	% Rural	Percentage of population living in a rural area.
